# System for reception and risk classification in obstetrics: a technical quality assessment*

**DOI:** 10.1590/1518-8345.3327.3330

**Published:** 2020-07-15

**Authors:** Rodolfo Cristiano Serafim, Milena Jamas Temer, Cristina Maria Garcia de Lima Parada, Heloisa Helena Ciqueto Peres, Clarita Terra Rodrigues Serafim, Rodrigo Jensen

**Affiliations:** 1 Universidade Estadual Paulista “Júlio de Mesquita Filho”, Faculdade de Medicina de Botucatu, Botucatu, SP, Brazil.; 2 Faculdade de Medicina, Hospital das Clínicas, Botucatu, SP, Brazil.; 3 Universidade de São Paulo, Escola de Enfermagem, São Paulo, SP, Brazil.

**Keywords:** Nursing Informatics, Health Information Systems, Technology Assessment, Biomedical, Emergency Care, Obstetric Nursing, User Embracement, Informática em Enfermagem, Sistemas de Informação em Saúde, Avaliação da Tecnologia Biomédica, Atendimento de Emergência, Enfermagem Obstétrica, Acolhimento, Informática Aplicada a la Enfermería, Sistemas de Información en Salud, Evaluación de la Tecnología Biomédica, Atención de Emergência, Enfermería Obstétrica, Acogimiento

## Abstract

**Objective:**

to assess the technical quality of a decision support system for reception and risk classification in obstetrics.

**Method:**

a methodological study of assessment of the system. 12 nurses and 11 information technology (IT) professionals were invited to evaluate the Obstetrics Reception and Risk Classification System (*Sistema de Acolhimento e Classificação de Risco em Obstetrícia*, SACR-O). Based on the standards of the International Organization for Standardization, the minimum number of evaluators and the characteristics to be evaluated were established: functional suitability, reliability, usability, performance efficiency, compatibility, safety, maintainability, and portability. The characteristics assessed should be given a ≥70% positive assessment to be considered suitable.

**Results:**

the characteristics assessed by the nurses and the IT professionals, respectively, were considered adequate: Functional suitability (97% and 98%), Reliability (91% and 94%), Usability (89% and 93%), Performance efficiency (97% and 98%), Compatibility (93% and 100%), and System security (95% and 97%). Maintainability (87%) and Portability (97%) were also evaluated by IT professionals.

**Conclusion:**

the technical quality of the SACR-O system was considered excellent by nurses and IT professionals.

## Abstract (pt)

**Objetivo:**

avaliar a qualidade técnica de um sistema de apoio à decisão para acolhimento e classificação de risco em obstetrícia.

**Método:**

estudo metodológico de avaliação de sistema. Foram convidados a avaliarem o Sistema de Acolhimento e Classificação de Risco em Obstetrícia (SACR-O) 12 enfermeiros e 11 profissionais de informática. A partir das normas da Organização Internacional de Normatização se estabeleceu o número mínimo de avaliadores e as características a serem avaliadas: adequação funcional, confiabilidade, usabilidade, eficiência de desempenho, compatibilidade, segurança, manutenibilidade e portabilidade. As características avaliadas deveriam ter avaliação positiva ≥70% para serem consideradas adequadas.

**Resultados:**

as características avaliadas por enfermeiros e profissionais de informática, respectivamente, foram consideradas adequadas, a saber: Adequação funcional (97% e 98%), Confiabilidade (91% e 94%), Usabilidade (89% e 93%), Eficiência de desempenho (97% e 98%), Compatibilidade (93% e 100%) e Segurança do sistema (95% e 97%). Por profissionais de informática foram também avaliadas as características de Manutenibilidade (87%) e Portabilidade (97%).

**Conclusão:**

a qualidade técnica do sistema SACR-O foi considerada como excelente por enfermeiros e profissionais de informática.

## Abstract (es)

**Objetivo:**

evaluar la calidad técnica de un sistema de soporte de decisiones para la recepción y clasificación de riesgos en obstetricia.

**Método:**

estudio metodológico de evaluación del sistema. Fueron invitados a evaluar el Sistema de Recepción y Clasificación de Riesgos en Obstetricia (SACR-O) 12 enfermeros y 11 profesionales de la informática. Con base en las normas de la Organización Internacional de Estandarización, se estableció el número mínimo de evaluadores y las características a evaluar: adecuación funcional, fiabilidad, usabilidad, eficiencia de desempeño, compatibilidad, seguridad, mantenibilidad y portabilidad. Las características evaluadas deberían tener una evaluación positiva ≥70% para considerarse adecuadas.

**Resultados:**

las características evaluadas por enfermeros y profesionales de la informática, respectivamente, se consideraron adecuadas, a saber: Adecuación funcional (97% y 98%), Fiabilidad (91% y 94%), Usabilidad (89% y 93%), Eficiencia de desempeño (97% y 98%), Compatibilidad (93% y 100%) y Seguridad del sistema (95% y 97%). Por los profesionales de informática también fueron evaluados las características de Mantenibilidad (87%) y Portabilidad (97%).

**Conclusión:**

la calidad técnica del sistema SACR-O fue considerada excelente por los enfermeros y los profesionales de la informática.

## Introduction

In 2011 the Ministry of Health (MoH) launched the *Rede Cegonha* (Stork Network) Program, which aimed to provide women and children with better health care and quality, with emphasis on actions to reduce maternal and infant mortality, in line with the National Policy for the Humanization of Attention and Management of the Unified Health System. In addition, in 2014, the MoH established the handbook for Reception and Risk Classification in Obstetrics (*Acolhimento e Classificação de Risco em Obstetrícia*, A&CRO), updated in 2017, to all obstetric urgency and emergency services in the country^(^ 1 ^-^ 4 ^)^.

The A&CRO handbook aims to provide guidance and standardization of conduct to health professionals who work in childbirth care services, in order to avoid problems in care that may culminate in unfavorable outcomes, as well as to enable qualified access and resolutive care timely for each case^(^ 4 ^)^.

The obstetrics risk classification is a decision support tool for the immediate identification of the pregnant woman’s severity, which ensures fast and safe care according to the risk potential and based on scientific evidence^(^ 4 ^-^ 6 ^)^.

The A&CRO protocol allows the pregnant woman to be classified for the medical care, according to the degree of urgency and from a clinical decision-making process, in which the maximum recommended waiting time to receive care is determined. There are five priority levels and each one corresponds to a maximum waiting time for medical care, namely: red (immediate care), orange (up to 15 min), yellow (up to 30 min), green (up to 120 min) and blue (not priority or referral according to agreement)^(^ 4 ^)^.

The A&CRO protocol is an important knowledge base to support obstetrics professionals’ decisions, as well as to generate positive impacts on care and error reduction. Thus, it is of great importance that instruments such as the A&CRO protocol are part of the routine of health care institutions and are available for use in electronic devices, integrated into the patient’s electronic record (PER), as a form of registration for later consultation and re-evaluation^(^ 7 ^-^ 8 ^)^.

In the information age, alternatives to health registering have been proposed in order to find strategies to store and qualify data registration, to build safer and more quality health care^(^ 9 ^)^.

The focus of the present study, the Obstetrics Reception and Risk Classification System (SACR-O) was developed in a university hospital in the interior of the state of São Paulo as a decision support system, built based on the A&CRO manual proposed by the MoH.

The system guides the nurse with structured questions and fixed values in their answers, based on the signs and symptoms presented by the pregnant woman. When finally filled, the system indicates the risk classification by color, as proposed by the MoH^(^ 4 ^)^.

SACR-O also offers a simulation database, allowing real-time training for undergraduate students, residents, and professionals of maternity hospitals for teaching or continuing education. The system is integrated into the PER, which favors the continuity of care and information security.

Considering the evaluation of health information systems, a systematic review study has shown that the best methods are those that follow the assumptions of the International Organization for Standardization (ISO) and the International Electrotechnical Commision (IEC), of the Brazilian Association of Technical Standards (*Associação Brasileira de Normas Técnicas*, ABNT) and the Brazilian Standard *(Norma Brasileira*, NBR) series, also with the certifications of computerized systems, which undergo rigorous testing by the Brazilian Society of Health Informatics (*Sociedade Brasileira de Informática em Saúde*, SBIS) together with the Federal Council of Medicine *(Conselho Federal de Medicina*, CFM)^(^ 10 ^)^.

From this perspective, this study aims to evaluate the technical quality of a decision support system for reception and risk classification in obstetrics.

## Method

This is a methodological study for the assessment of a system. Methodological studies are characterized by the development, validation, and evaluation of research tools and methods^(^ 11 ^)^.

The research was conducted in the maternity ward of a public hospital in the interior of the state of São Paulo. This is a tertiary care level general hospital, which has a hospital information system (MV Sistemas^®^) that gathers the clinical and assistance information of all the care services.

SACR-O was developed to allow for the classification of pregnant women to be recorded in the PER, with the purpose of maintaining data security and control. Thus, it is necessary that the professional who performs the service login into the system with the user and password. When entering the system, one has access to the A&CRO stages and, for a new care service, they can access areas such as opening the appointment, updating the pregnant woman’s registration, specialized professional care, medication administration, laboratory and image exams; even the end of the appointment with discharge or referral to the obstetric center.

To participate in the technical quality evaluation of SACR-O, information technology (IT) professionals with training in the areas of analysis and development of systems were invited, as well as nurses and residents in obstetric nursing of the institution where the study was conducted. For nurses and residents, it was a criterion for inclusion to have carried out the A&CRO at least once in service.

ISO/IEC standards were respected, namely: ISO/IEC 25040:2011^(^ 12 ^)^, which indicate the minimum number of eight evaluators, and the ISO/IEC 25010:2011^(^ 13 ^)^ which proposes the evaluation of the technical quality of the software by eight characteristics: functional suitability, reliability, usability, performance efficiency, maintainability, portability, safety, and compatibility. Each characteristic is composed of sub-characteristics, totaling 31 sub-characteristics. Given the technical specificities of the maintainability and portability characteristics, these were evaluated only by IT professionals, as pointed out in the literature^(^ 14 ^)^.

The quality when using a software, or usability, is composed of characteristics and sub-characteristics related to the result of interaction when a product is used in a context of specific use. This system model is applicable to the human-computer interaction system, including using computer systems and using software products. The product quality model, or technical quality, is composed of characteristics and sub-characteristics related to the software’s static properties and the system’s dynamic properties^(^ 13 ^)^.

The questionnaire used in this study to assess the technical quality of the system was built on an instrument proposed in a previous study^(^ 14 ^)^.

The specialists, IT professionals and nurses, accessed the system with guidelines on the evaluation process and received a handbook with information on the screen structures and detailed specifications for each item. A simulation environment was made available to the specialists, with access user and password to the system and a clinical case, set up by the authors of the study. The evaluation occurred individually, without interference by the researchers.

The experts evaluated each characteristic and sub-characteristic using one of the following options: agree (Level A); disagree - Justify (Level D); and not applicable (Level NA).

Level A means that the system has met the requirement; Level D, that it did not meet the requirement, thus needs justification for improvements; and Level NA, that they could not assess or it is not applicable.

To obtain the sum of the values of the characteristics and sub-characteristics the data were analyzed as proposed in the ISO/IEC 14598-6:2004 norm, using the following formula:

$$\mathrm{Vc}=\frac{\sum \mathrm{Vsc}}{\mathrm{nsc}}\;\mathrm{Vcs}=\frac{\sum m}{\left(n-\mathrm{nd}\right)}$$

Vc = mean value of the characteristic

Vsc = mean value of the sub-characteristic

nsc = the number of the sub-characteristic

m = 1, if the answer is positive, otherwise it is 0

nd = the number of discarded questions

Thus, to obtain the values of its characteristics and percentages, the following formula was applied:

$$\mathrm{Vc}=\frac{\sum \mathrm{Vsca}}{\left(a+d\pm \mathrm{na}-\mathrm{na}\right)}\times 100$$

Vc = mean value of the characteristic

Vsca = the value of the sub-characteristic with adequate answers

a = adequate answer

d = deficient answer

na = not applicable answer ^(^ 15 ^)^

In the evaluation of the results, an adapted scale was used with expected values for each characteristic and sub-characteristic; they should be above 70% to be considered satisfactory^(^ 14 ^)^. The categories and subcategories were evaluated with cutoff rates for weak (25%), regular (50%), good (75%) and excellent (100%)^(^ 15 ^)^.

The research was approved by the Research Ethics Committee of the Botucatu Medical School of the “Júlio de Mesquita Filho” State University of São Paulo (CAAE: 83484518.5.0000.5411).

## Results

In the results’ presentation, it was decided to separate the assessment of IT professionals and nurses as the training of these assessors may lead to different criteria in the assessment, according to their field. The characterization of the evaluators is described in Table 1.

**Table 1:** Characterization of evaluators, IT professionals (n=11) and nurses (n=12). Botucatu, SP, Brazil, 2018

Characteristics	IT professionals Mean (sd)	n (%)	Nurses Mean (sd)	n (%)
Age (years old)	40.4 (8.9)		27.9 (8.3)	
**Professional practice** (years)	16.1 (6.9)		4.9 (8.4)	
**Degree**				
- Undergraduate degree		3 (27.3)		4 (33.3)
- Specialization		8 (72.7)		8 (66.7)
**Type of professional practice**				
- Support				
- Teaching/Research				11(91.7)
- Systems Analyst		9 (81.8)		1(8.3)
- Programmer		1 (9.1)		
- IT Manager		1 (9.1)		

In comparison, the nurses were younger and had fewer working years. Regardless of the professional category, most participants had specialist titles (Table 1).

**Figure 1:**
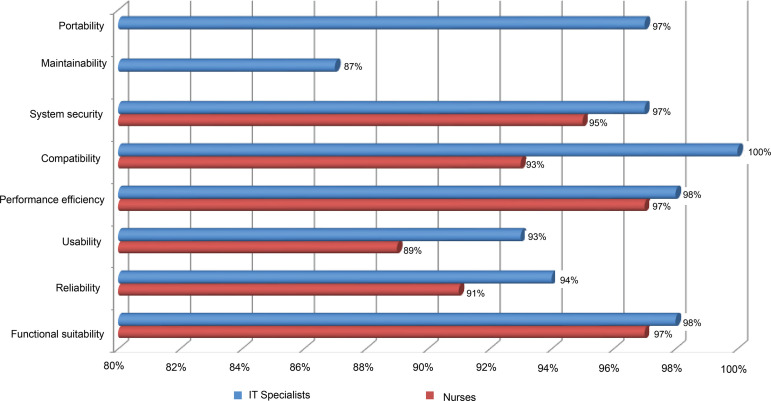
Evaluation of the technical quality’s characteristics of the system by IT professionals and nurses. Botucatu, SP, Brazil, 2018

The assessment of the technical quality’s characteristics is described in Figure 1.

All the technical quality categories evaluated were considered adequate, with an evaluation of over 70%.

As for the evaluation of the sub-characteristics, at least one in each characteristic obtained 100% adequacy. Only the sub-characteristic accessibility, of the usability characteristic, obtained an evaluation lower than 70% among IT professionals (Table 2).

**Table 2:** Evaluation of the sub-characteristics of the system by IT professionals (n=11) and nurses (n=12). Botucatu, SP, Brazil, 2018

Characteristics	Sub-characteristics	IT professionals %	Nurses %
1. Functional suitability	1.1 Functional integrity	100	92
	1.2 Functional correction	100	100
	1.3 Functional aptitude	91	100
2. Reliability	2.1 Maturity	100	86
	2.2 Fault tolerance	86	100
	2.3 Recoverability	89	80
	2.4 Availability	100	100
3. Usability	3.1 Recognition of suitability	95	100
	3.2 Learnability	100	81
	3.3 Operability	95	74
	3.4 Accessibility	40	80
	3.5 Error protection	100	100
	3.6 Interface aesthetics	100	96
4. Efficiency and performance	4.1 Time	95	92
	4.2 Resources	100	100
	4.3 Capacity	100	100
5. Compatibility	5.1 Interoperability	100	90
	5.2 Coexistence	100	100
6. Safety	6.1 Confidentiality	100	100
	6.2 Integrity	93	88
	6.3 Non-repudiation	100	92
	6.4 Accountability	100	100
	6.5 Authentication	100	100
7. Maintainability	7.1 Analyzability	82	-
	7.2 Modifiability	73	-
	7.3 Testability	100	-
	7.4 Modularity	91	-
	7.5 Reusability	91	-
8. Portability	8.1 Adaptability	91	-
	8.2 Capacity to be installed	100	-
	8.3 Ability to replace	100	-

## Discussion

Apart from organizing the flow of urgency and emergency services, SACR-O favors professionals’ respect to the A&CRO protocol of the MS^(^ 4 ^)^, ensuring ethical and adequate care for pregnant women.

SACR-O has shown a satisfactory suitability rate in all characteristics, over 87%. The characteristics functional suitability and performance efficiency obtained 97% of approval among nurses and 98% among IT professionals. The suitability of these characteristics corresponds to the satisfaction of the users, in order to achieve the objectives of using the system with precision and integrity. Performance efficiency is linked to system response time, considered in this study as satisfactory for both categories of SACR-O in these items^(^ 14 ^,^ 16 ^)^.

Reliability represents how much the system can deliver the level of performance when exposed for a given time and circumstances, such as being able to perform its tasks reliably with limited resources. For the evaluators, this characteristic was over 90%, which reflects users’ confidence in the system. We stress that the reliability of computerized systems has shown higher rates when compared to manual systems^(^ 9 ^,^ 14 ^)^.

The usability characteristic has been extensively studied in health information system evaluations, as it evaluates the accuracy of the system and the efficiency to users. It often identifies complaints such as slow system response time, too much information on the screen, and changing the workflow^(^ 17 ^-^ 18 ^)^.

As for the sub-characteristic accessibility, in the evaluation by IT professionals, the expected value was not reached, being considered as regular. However, it was considered excellent by the nurses. The suggestion pointed out by IT professionals to accessibility was to insert a magnifying glass resource, for users with visual difficulty.

The assessment below expected values reflects the need to reassess system requirements. Due to the time for the development of the study, it was not possible to reevaluate this sub-characteristic after adjustments. Other studies^(^ 18 ^)^ also describe results with fragility in the evaluation of accessibility, related to the resolution and visibility of the screen. Some authors^(^ 19 ^)^ recommend that in order to serve people with disabilities, especially visually impaired ones, health information systems should present visually clear screens, with organized information and easy-to-view images.

Also regarding usability, the sub-characteristic error protection obtained 100% adequacy among nurses and IT professionals, which demonstrates that SACR-O offers security in the registration of information.

In the compatibility characteristic, which includes the sub-characteristics interoperability and coexistence, IT professionals evaluated 100% adequacy in both sub-characteristics, being the only characteristic with the maximum consensus value; nurses indicated 90% adequacy for interoperability and 100% for coexistence. This characteristic reflects the ability to exchange information with other systems, for example, the exchange of information with the PER^(^ 14 ^,^ 17 ^)^. Interoperability is a concern for health information systems since the lack of integration between systems can compromise patient care^(^ 11 ^,^ 14 ^,^ 17 ^)^.

The safety characteristic obtained the criterion of excellence in three of its sub-characteristics by the evaluators. This is one of the main features to be considered today since health information systems store a large amount of patients’ information and are essential for continuous care. Login and password authentication methods are crucial, as well as the need for effective backup systems, for the ability to store information and reuse it in case of loss or downtime of the main system^(^ 20 ^-^ 21 ^)^.

The maintainability characteristic, which aims to show the necessary efforts to make specific modifications, and the portability characteristic, that demonstrates the capacity of the system to be transferred to another operational environment, presented rates of adequacy corresponding to data from previous studies, with adequacy higher than 70%, considered satisfactory in this requirement^(^ 14 ^,^ 22 ^)^.

Systems for risk classification of patients in the hospital urgency and emergency services have been widely used. SACR-O, developed in accordance with the MoH A&CRO protocol, is relevant because it strengthens MoH policies, favors the agility of services and consequently the humanization of assistance. It is important to note that the adequate technical quality of health information systems is today considered a factor of patient safety^(^ 23 ^)^.

We consider as a study limitation the inadequacy of the system in terms of the sub-characteristic of accessibility in time to be re-evaluated by IT professionals.

The limited number of publications on health information systems evaluation is noteworthy. This review^(^ 24 ^)^ demonstrated the need for further research and dissemination, with emphasis on the use of information systems in nursing.

We highlight the importance of this study to support the decision of managers and developers in building and acquiring reliable systems that improve the quality of health care.

## Conclusion

The use of information and communication technologies for decision support has the potential to improve and enhance health care. This study aimed to evaluate the technical quality of SACR-O and the results obtained showed adequacy higher than 87% in all characteristics evaluated, obtaining an excellent criterion.

In the evaluation of the system, the software quality metrics indicated by ISO/IEC were used, with a set of six to eight characteristics, composed by sub-characteristics.

The results achieved from the evaluation indicate that SACR-O has proved adequate in all characteristics. We stress that at least one sub-characteristic, of each characteristic evaluated, reached 100% of adequacy: Functional correctness of the Functional suitability characteristic; Availability of the Reliability characteristic; Error protection of the Usability characteristic; Resources and Capability of the Efficiency and performance characteristic; Coexistence of the Compatibility characteristic; and Confidentiality, Accountability, and Authentication of the Security characteristic.

SACR-O complies with the A&CRO handbook proposed by the MoH, thus showing potential and safety in the information of the reception and risk classification of pregnant women. With this, it can support nurses in decision making and in the flow of care.
